# Wildlife as Reservoirs of *Encephalitozoon Cuniculi* and *Encephalitozoon Hellem and* Molecular Genotyping of *Encephalitozoon* spp. in Small Mammals in the Czech Republic

**DOI:** 10.1007/s11686-024-00920-0

**Published:** 2024-10-02

**Authors:** Eva Bártová, Jiřina Marková, Alena Žákovská, Zuzana Čadková, Marie Budíková

**Affiliations:** 1 Department of Biology and Wildlife Diseases, Faculty of Veterinary Hygiene and Ecology, University of Veterinary Sciences Brno, Palackého tř. 1946/1, Brno, 61242 Czech Republic; 2 Department of Experimental Biology, Faculty of Science, Masaryk University, Kamenice 5, Brno, 625 00 Czech Republic; 3 Department of Biology, Faculty of Education, Masaryk University, Kamenice 753/5, Brno, 625 00 Czech Republic; 4 Department of Zoology and Fisheries, Faculty of Agrobiology, Food and Natural Resources, Czech University of Life Sciences Prague, Kamýcká 129, Prague Suchdol, 165 00 Czech Republic; 5 Department of Mathematics and Statistics, Faculty of Science, Masaryk University, Brno, Czech Republic

**Keywords:** Encephalitozoonosis, Wildlife, Urban area, Zoonosis, Genotyping

## Abstract

**Purpose:**

Parasites of genus *Encephalitozoon* are well known pathogens of domestic animals however less attention was paid to its spread among wildlife that can play an important role of reservoir of infection. The aim of the study was to conduct molecular detection and genotype characterization of *Encephalitozoon* spp. in wild small mammals trapped in localities both near to and at a large distance from residential areas.

**Methods:**

In total, 300 wild small mammals (274 Rodentia and 26 Eulipotyphla) were trapped in 41 localities of the Czech Republic and tested by nested PCR for *Encephalitozoon* spp.

**Results:**

The DNA of *Encephalitozoon* spp. was proved in tissues (brain or liver) of 11% (32/300) of animals. There was a statistically significant difference (*p* < 0.001) in positivity among animal species with the most infected species *Micromys minutus* (50%, 4/8) and *Myodes glareolus* (17%, 9/53). There was also statistically significant difference (*p* < 0.001) between localities with the higher positivity (29%, 12/42) in localities near to residential areas, compared to localities with a large distance from residential areas (8%, 20/258). Sex and age of wild small mammals did not have effect on their positivity. Genotyping analysis revealed *E. cuniculi* genotype II in 22 samples and *E. hellem* genotype 1 A in one sample.

**Conclusion:**

This study brings new information on the molecular characterization of *Encephalitozoon* spp. isolated from wild small mammals trapped in two different areas (localities in near to residential areas and localities with a large distance from residential areas).

## Introduction

*Encephalitozoon* spp. is a microsporidian obligate intracellular spore-forming parasite and is considered to be one of the most common microsporidian parasites in humans [1]. Encephalitozoonosis is rare in healthy people but can be a complication in patients with weakened immune systems [2–5]. The species *Encephalitozoon cuniculi*, *Encephalitozoon intestinalis* and *Encephalitozoon hellem* were detected in various birds and mammals [6] with a high prevalence of *Encephalitozoon* spp. in some of the domestic animals. Wild animals can also be infected and can serve as reservoirs of several infections including zoonotic diseases [7]. The usage of molecular methods helps to detect and differentiate strains of *Encephalitozoon* spp.

Didier et al. [8] described 3 different genotypes (I, II, and III) of *E. cuniculi* strains with different number of 5′-GTTT-3′ repeats in the internal transcribed spacer (ITS) region of the ribosomal RNA gene. More recently, a novel genotype IV was identified in a human patient [9]. The types of *E. cuniculi* are sometimes referred as the “rabbit strain” for type I, the “mouse strain” for type II, the “dog strain” for type III, and the “human strain” for type IV in association to the host from which they were isolated. Although each strain has a preferential host species, it is known that *E. cuniculi* has a low host specificity. For example, genotype III (“dog strain”) was detected also in wild small rodents [10, 11]. The main host of *E. hellem* are humans, but it has been found also in birds. Correspondingly, Mathis et al. [12] identified strain variation of *E. hellem* based on the sequence of the ITS of the ribosomal DNA (rDNA) showing the ITS sequence seems a valuable marker.

Free-living small mammals, mainly rodents, are known carriers and reservoirs of more than 60 diseases with zoonotic potential. Thanks to their adaptive way of life, they can be found both in the wild and in area heavily populated with humans and thus can represent the risk of infection for domestic animals and human. Since the sylvatic life cycle of *Encephalitozoon* spp. is not yet fully understood, the aim of the study was to conduct molecular detection and genotype characterization of *Encephalitozoon* spp. in wild small mammals trapped in localities both near to and at a large distance from residential areas.

## Materials and Methods

### Sampling

**Fig. 1:**
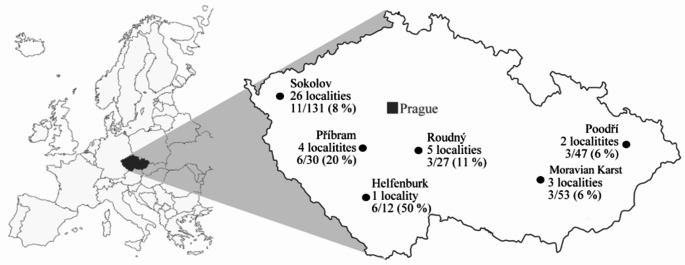
Map showing 41 localities of 6 areas (4 areas: Sokolov, Příbram, Roudný, and Helfenburk in Bohemia and 2 areas: Poodří Protected Landscape Area and the Moravian Karst in Moravia) in the Czech Republic, where 300 wild small mammals were trapped for detection *Encephalitozoon* spp. by nested PCR

Wild small mammals were trapped using the snap and life traps in 41 different localities of the Czech Republic (Fig. 1) during two trapping sessions (June and September) in the years 2015 and 2016.

In Bohemia (western part of the Czech Republic), the trapping was done in 36 localities of 4 areas (Helfenburk, Příbram, Roudný, and Sokolov). In Helfenburk and Příbram, the trapping plots were situated near to residential zones, recreational areas, and sports facilities. On the contrary, in Sokolov and Roudný, the trapping plots were located significantly further from residential areas in grasslands, meadows, forests, wetlands, and spoil heaps. At each trapping site, 25 traps were set for 3 consecutive nights in a standard trapping grid or lines (based on the plots’ characteristics) with a 5-metre distance trap-trap.

In Moravia (eastern part of the Czech Republic), the trapping was done in 5 localities of 2 protected landscape areas (the Moravian Karst and Poodří Protected Landscape Area), further from residential areas, in wet meadows, hornbeam, and oak forests. The traps were placed on the ground in a line, with 7-metre spacing between them.

The researchers involved in the sampling were experienced in safety protocols during animal trapping and handling and followed the conditions of the long-term experimental project (Ethical Approval Statement). The basic principles of biosafety to protect persons and the environment were respected. The animals were necropsied in an isolated zone of the laboratory by personnel dressed in laboratory coats, gloves, eye and face protection and 3 M respirators.

In total, 300 trapped animals (274 Rodentia and 26 Eulipotyphla) of different species, sex, and age (adult, sub-adult, and juvenile) were analyzed for the detection of *Encephalitozoon* spp. (Table 1). Tissue samples (brain or liver) collected during autopsy were frozen at – 20 ºC until DNA isolation.

**Table 1:** Detection of *Encephalitozoon* spp. by nested PCR in wild small mammals trapped in the Czech Republic

Characteristic	Bohemia	Moravia	Total	*P*-value
Species				0.0015*
*Apodemus agrarius*	-	1/20 (5%)	1/20 (5%)	
*Apodemus flavicollis*	1/48 (2%)	0/20	1/68 (1%)	
*Apodemus sylvaticus*	9/49 (18%)	0/20	9/69 (13%)	
*Apodemus uralensis*	-	0/2	0/2	
*Micromys minutus*	4/8 (50%)	-	4/8 (50%)	
*Microtus agrestis*	0/5	-	0/5	
*Microtus arvalis*	4/45 (9%)	1/3 (33%)	5/48 (10%)	
*Mus musculus*	0/1	-	0/1	
*Myodes glareolus*	6/33 (18%)	3/20 (15%)	9/53 (17%)	
*Neomys anomalus*	0/1	-	0/1	
*Sorex araneus*	2/10 (20%)	1/15 (7%)	3/25 (12%)	
**Sex**				0.2618
Female	16/100 (16%)	3/50 (6%)	19/150 (13%)	
Male	10/100 (10%)	3/50 (6%)	13/150 (9%)	
**Age**				0.0910
Juvenile	5/21 (24%)	2/39 (5%)	7/60 (12%)	
Subadult	7/68 (10%)	-	7/68 (10%)	
Adult	14/111 (13%)	4/19 (21%)	18/130 (14%)	
Not known	-	0/42	0/42	
**Proximity to residential area** ^**a**^				0.0001*
Localities with close distance	12/42 (29%)		12/42 (29%)	
Localities with large distance	14/158 (9%)	6/100 (6%)	20/258 (8%)	
**Total**	**26/200 (13%)**	**6/100 (6%)**	**32/300 (11%)**	

*P-value ≤ 0.05 statistically significant difference, ^a^Proximity to residential areas: close distance (Helfenburk and Příbram), large distance (Roudný, Sokolov, Moravian Karst, Poodří). Two genotypes were proved: *E. cuniculi* genotype II in 22 samples (in 1 *A. agrarius*, 4 *A*. *sylvaticus*, 8 *C. glareolus*, 5 *M. arvalis*, 2 *M. minutus* and 2 *S. araneus*); *E. hellem* genotype 1 A in 1 individual (*A. sylvaticus*)

### Molecular Methods

The DNA was isolated by DNeasy^®^ Blood & Tissue Kit (Qiagen, Hilden, Germany) following producer instructions. Before that, pieces (≤ 25 mg) of brain or liver (when brain was not available) tissues were homogenized by MagNA Lyser Instrument (Roche, Basel, Switzerland), using ceramic marbles. The samples were examined by nested PCR amplifying rDNA internal transcriber spacer region (ITS), with the use of two pairs of microsporidia-specific MSP1, MSP2A, MSP3 and MSP4A primers [13]. The PCR mixture contained 12 µl of commercial premix PPP master mix (Top-Bio s.r.o., Prague, Czech Republic), 6 µl of PCR grade water, 1 µl of each primer and 5 µl of DNA. A positive control (DNA isolated from spores of *E. cuniculi*) and a negative control (PCR grade water, Top-Bio s.r.o.) were included in the PCR reaction. The PCR protocol for both steps included an initial denaturation at 92 °C for 2 min, followed by 35 cycles of denaturation at 92 °C for 1 min, annealing at 59 °C for 1 min, and extension at 72 °C for 1.5 min, with a final extension step at 72 °C for 5 min. After electrophoresis on 1.5% agarose gel with Midori Green stain, animals were evaluated to be positive, when tissue (brain or liver) was positive. Positive PCR products were purified (Gel/PCR Fragments Extraction Kit, Geneaid, New Taipei City, Taiwan) and sent for sequencing (Macrogen, Amsterdam, the Netherlands). Final sequences were edited by Staden Package Programs (Pregap4, Gap4) and compared with sequences in GenBank using BLAST (Basic Local Alignment Search Tool).

### Statistical Analysis

The results were statistically analysed with Pearson’s chi-square test for independence, using STATISTICA Cz 12 [14]. We tested the null hypothesis that a prevalence of *Encephalitozoon* spp. does not differ among species, sex, age, and areas of trapping (proximity to residential area). The differences were considered statistically significant if the *p*-value was < 0.05. In the case of a statistically significant difference of positivity in some of the variables, the Scheffé’s multiple comparison method [14] was subsequently applied to identify a statistically significant difference between pairs of animal species. Subsequently, the odds ratio (OR) of chances for these pairs was calculated. The results were presented as percentages with 95% confidence intervals (CI).

## Results

The DNA of *Encephalitozoon* spp. was proved by nested PCR in 11% (32/300) of animals, with 13% (26/200) in animals trapped in Bohemia and 6% (6/100) in animals trapped in Moravia, without statistical difference (*p* > 0.05) in these two parts of the Czech Republic (Table 1). The Scheffé’s multiple comparison method showed a statistically significant difference (*p* < 0.0015) in prevalence of *Encephalitozoon* spp. between species, specifically in *Apodemus flavicollis* (1%) and *Micromys minutus* (50%) (OR = 69. 95% CI: 6.0 -747.7). The prevalence of *Encephalitozoon* spp. statistically differed (*p* < 0.05) depending on the areas of trapping, with a higher prevalence (29%, 12/42) in areas close to residential areas, compared to the prevalence (9%, 20/258) in areas at a large distance from residential areas. There was no statistically significant difference (*p* > 0.05) in prevalence between the sex and age categories of animals.

Analysis of the genotypes by BLAST search in the GenBank database showed the highest homology with *E. cuniculi* genotype II in 22 of 32 positive samples (1 isolated from *Apodemus agrarius*, 4 from *Apodemus sylvaticus*, 8 from *Myodes glareolus*, 5 from *Microtus arvalis*, 2 from *Micromys minutus*, and 2 from *Sorex araneus*). Sixteen of these samples were from animals trapped in Bohemia (2 from Helfenburk, 4 from Příbram, 2 from Roudný and 8 from Sokolov), and six were from animals trapped in Moravia (3 from The Moravian Karst and 3 from Poodří). One sample isolated from juvenile female *A. sylvaticus* trapped in Bohemia (Helfenburk) was characterised as *Encephalitozon hellem* genotype 1 A. Genotyping of 9 samples was not completed.

## Discussion

Wild animals living in urban or suburban areas are often monitored as reservoirs of various pathogens. In our study, molecular methods confirmed the presence of *Encephalitozoon* spp. DNA in the brain or liver of 11% of wild small mammals. In a previous study, the DNA of *E. cuniculi* was detected by PCR in brain of 6.5% wild small mammals from Austria [15] and affection of the central nervous system was also described in rabbits as one of the most common symptoms of *E. cuniculi* disease [5, 16]. In other studies from Central Europe, different tissue samples were used for detection of *Encephalitozoon* spp. For example, Perec-Matysiak et al. [11] examined by nested PCR spleen and faecal samples of wild small mammals trapped in nature reserves and suburban recreational areas in Slovakia, Poland, and the Czech Republic. They found *Encephalitozoon* spp. in 15% of animals, which was only slightly higher than the positivity in our study. Sak et al. [17] proved *Encephalitozoon* spp. by nested PCR in faecal samples of 25% wild rodents trapped across the Czech Republic-German border. They showed only a slight correlation between positivity and the proximity to residential areas that is in contrast to our results. Danišová et al. [18] proved *E. cuniculi* and *E. intestinalis* by real-time PCR in faecal samples of 0.4% wild small mammals from Slovakia.

The trapping localities in our study had different characteristics in terms of proximity to residential areas, with statistically higher *Encephalitozoon* spp. positivity in animals trapped in near to residential areas. The most often infected animal species in our study was *M. minutus*, with all animals of this species having been trapped close to residential areas (proximity to a football stadium and adjacent family houses in Příbram). In Bohemia, *Encephalitozoon* spp. was detected in 13% of animals trapped in urban areas, whereas a lower positivity of 6% was observed in animals trapped in protected landscape areas in Moravia, where there is less frequent contact with humans or domestic animals.

Based on molecular techniques, the presence of 4 different genotypes of *E. cuniculi* (genotypes I-IV) was previously confirmed [8, 9]. Genotyping analysis of positive samples from our study showed that 69% (22/32) were *E. cuniculi* genotype II and 3% (1/32) *E. hellem* genotype 1 A. *E. cuniculi* genotype II was also proved in 93% of wild rodents from Slovakia, Poland, and the Czech Republic, while genotypes I and III were proved only in 1.5% and 6% of animals, respectively [11]. In contrast, Sak et al. [17] proved genotype I to be more frequent (58%) compared to genotype II (42%) in wild small mammals from the Czech Republic. Based to the previous studies, it seems that genotype II is more frequent in wildlife reservoirs compared to other genotypes, nevertheless, infection with this genotype was also described in humans e.g. in 8% of renal transplant recipients and in 1% of patients with various respiratory diseases [19]. Despite the fact that *E. hellem* genotype 1 A is typical for birds [6], this genotype was proved in material from human immunodeficiency virus-positive patients [20] and also in 9% of 2 subspecies of the house mouse trapped in the Czech Republic and Germany near or within residential areas or stables [17]. This points to the fact that host specificity is not strictly limited to birds. Central Europe, and especially the Czech Republic, is among the best explored areas concerning the number of studies focused on the spread of *E. cuniculi* infection in wildlife [6]. Further research in this country is essential to explain the method of transmission of infection in wildlife, which has not yet been fully understood.

Based on our results, wild small mammals can play a crucial role as a reservoir in the transmission of *Encephalitozoon* spp. infection. They can spread *Encephalitozoon* spp. through sympatric rodents to the domestic animals, but they may also spillover the infection from domestic animals [21]. This could pose a threat to domestic animals and to humans, who might become infected with *Encephalitozoon* spp. from food and water contaminated with spores or through the consumption of infected farm animals. Moreover, according to a laboratory experiment, spores of *E. cuniculi* genotype II may remain infective even after fermentation at 24 °C for 48 h [22]. This contributes to the risk of alimentary infection in humans after ingestion of uncooked meat products from domestic animals containing spores of *E. cuniculi*.

It seems that there was a sufficient scope in terms of the diversity of localities with four areas in Bohemia, and two in Moravia including also the area in warmer southern Moravia. A limitation of the study could be the sample size (*n* = 300) and the result might be partly influenced by the sequencing results, when just 22 of 32 positive samples (69%) were successfully sequenced. The rest positive samples could distort the ratio of occurrence of genotype II to genotype I, however, it is evident that genotype II would prevail.

## Conclusion

The *Encephalitozoon* spp. is parasite present worldwide and circulates in both animal and human populations. Given its high environmental resistance, low host specificity, and potential to cause serious disease or even death in both animals and humans, it is important to study this pathogen within a One Health framework. We proved higher positivity in localities near to residential areas compared with other with a large distance. Even more, genotyping analysis revealed *E. cuniculi* genotype II and *Encephalitozon hellem* genotype 1 A. The results of our study brought new information for better understanding the sylvatic life cycle of *Encephalitozoon* spp.

## Data Availability

No datasets were generated or analysed during the current study.
